# Process evaluation of implementation of the early stages of a whole systems approach to obesity in a small Island

**DOI:** 10.1186/s12889-024-18876-1

**Published:** 2024-05-22

**Authors:** Brittney MacKinlay, Kate Heneghan, Alexandra J. Potts, Duncan Radley, George Sanders, Ian F. Walker

**Affiliations:** 1 UK Overseas Territories Programme, Department of Health and Social Care, Office for Health Improvement and Disparities, 39 Victoria Street, London, SW1H 0EU UK; 2 Health Promotion Lead, St Helena Government, St Helena, UK; 3 Carnegie School of Sport, Leeds Beckett University, Leeds, UK

**Keywords:** Process evaluation, Obesity, Small island developing states, Whole systems approach, Complex interventions

## Abstract

**Background:**

The small Atlantic island of St Helena is a United Kingdom Overseas Territory (UKOT) with a high prevalence of childhood obesity (over a quarter of 4–5 and 10–11 year olds) and, anecdotally, adulthood obesity and its associated health detriments. St Helena have taken a whole systems approach to obesity (WSAO) to address the issue. A WSAO recognises the factors that impact obesity as a complex system and requires a ‘health in all policies’ approach. UK academic and public health technical support was provided to the local St Helena delivery team. This process evaluation sought to explore the early stages of the WSAO implementation and implications for the transferability of the approach to other small island developing states and UKOT.

**Methods:**

Data was collected via eight semi-structured interviews, paper based and online surveys, and document analysis. Thematic analysis was used to analyse the data.

**Results:**

The analysis identified three factors which aided the first phase of WSAO implementation: (1) senior leaders support for the approach; (2) the academic support provided to establish and develop the approach; and (3) effective adaptation of UK Government resources to suit the local context. Key challenges of early implementation included: maintaining and broadening stakeholder engagement; limited local workforce capacity and baseline knowledge related to obesity and systems thinking; and limited capacity for support from the UK-based academic team due to contract terms and COVID-19 restrictions.

**Conclusions:**

Early stages of implementation of a WSAO in a UKOT can be successful when using UK’s resources as a guide and adapting them to a small island context. All participants recommended other small islands adopt this approach. Continued senior support, dedicated leadership, and comprehensive community engagement is needed to progress implementation and provide the foundation for long-term impact. Small island developing states considering adopting a WSAO should consider political will, senior level buy-in and support, funding, and local workforce knowledge and capacity to enable the best chances of successful and sustainable implementation.

**Supplementary Information:**

The online version contains supplementary material available at 10.1186/s12889-024-18876-1.

## Background

Obesity is of great global public health concern [1] and one that is a challenge in many small islands, such as St Helena. St Helena is a small island located in the South Atlantic Ocean and is part of the UK Overseas Territory (UKOT) of St Helena, Ascension, and Tristan da Cunha. St Helena has a population of 4,534 [2]. Obesity is associated with reduced life expectancy [3, 4] and, particularly in adulthood, is a risk factor for chronic diseases such as cardiovascular disease, type 2 diabetes, at least 12 kinds of cancer [5], liver and respiratory disease [6], and some mental health conditions [7]. While BMI data for the general adult population is not available among St Helena adult residents (Saints), over one in three older adults (65–79 years) have diabetes, and one in two adults have high blood pressure [8]. Despite exact prevalence numbers being unavailable, in 2018 the St Helena government identified obesity as a priority health challenge to address (along with the high prevalence of NCDs) in their Strategic Framework for Health Promotion [9].Childhood obesity is associated with obesity in later life as well as premature death and disability [1]. Moreover, there are acute issues with childhood obesity such as breathing difficulties, increased risk of fractures, hypertension, early markers of cardiovascular disease, insulin resistance and psychological effects [1]. On St Helena, over a quarter of 4–5 year olds and 10–11 year olds were living with overweight or obesity in 2021 [10]. Preventing and addressing obesity in childhood seeks to break the intergenerational nature of obesity [11]. As such, it is important work is done to address a reduction in obesity at all stages of the life cycle.

In response to the increasing awareness of the complexity of many public health problems including obesity, a whole systems approach (WSA) has become a promising tactic [12]. Obesity is a complex, multi-causal problem with no one single solution. Addressing such an entrenched issue requires a long-term, system-wide approach that needs co-ordinated action across a broad range of disciplines and stakeholders, is tailored to local needs, and works across the life course [13]. A WSA to obesity epitomises a ‘Health in All Policies’ [14] approach and works with communities and stakeholders to both understand the problem and to support the identification and testing of solutions. A WSA can offer a sustainable, collaborative, community centred approach to address the complex problem of obesity whilst also having a positive impact on other local agendas, such as employability and productivity and reduced demand for social care. The World Health Organization’s (WHO) “Acceleration Plan to Stop Obesity” advocates for a multisector approach to address obesity [15].

United Kingdom (UK) Government guidance on implementing a whole systems approach to obesity (WSAO) provides local authorities in England with a practical approach to implementation [16]. This guidance includes a six-phase step by step process and a comprehensive range of supporting resources including an action mapping tool, network analysis tool, and guidance on system mapping. A mixed method evaluation undertaken in seven local authorities in England, indicated several short-term impacts [17]including: commencement of mindset changes and a move towards systems thinking; increased number of engaged partners; increased knowledge and understanding of systems science and obesity amongst partners and indicators of shifts towards targeting wider determinants of health rather than individual lifestyle factors. A process evaluation of seven local areas in Scotland implementing a WSAO found that the UK Government’s guidance helped to establish the approach by providing a well-structured, process-led framework and supported the development of a shared understanding and vision [18].

In 2021 St Helena began implementation of a WSAO using the UK guidance [16] alongside technical support (capacity building in systems thinking and methods and advice on implementation) from a team of UK public health experts. This provided the opportunity to evaluate the implementation of this approach. The clear need for such evaluation was highlighted in a recent systematic review, where the authors stated “evidence of how to operationalise a whole systems approach to address public health problems is still in its infancy” and “evidence highlights the limited progress that has been made in the practical implementation and evaluation of WSAs to public health issues to date” [13]. Therefore, this paper aims to present the first process evaluation of WSAO implementation on a small island setting.

## Methods

### Whole system approach to obesity

**Fig. 1:**
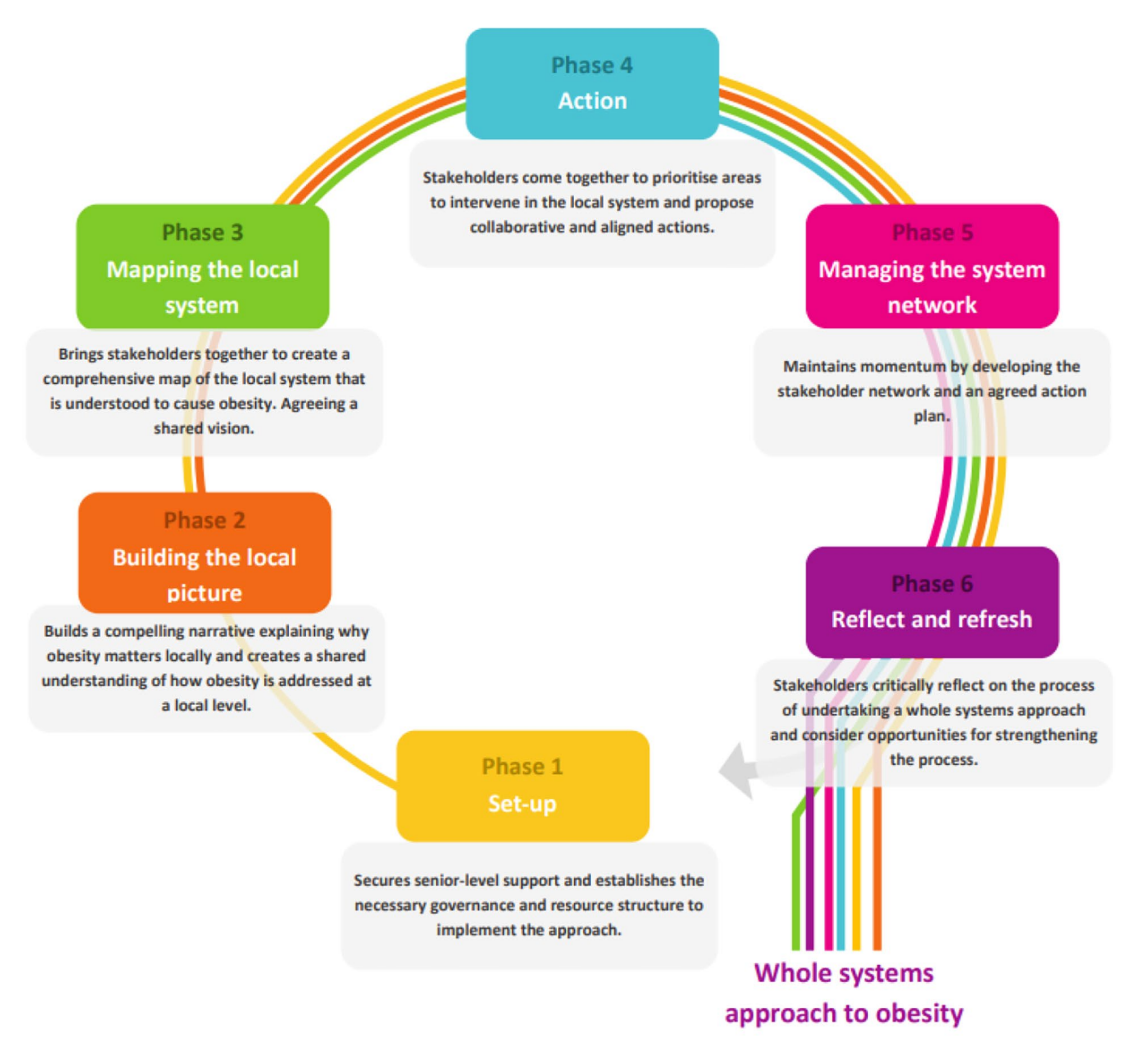
: Process for implementing whole systems approach to obesity [16]

The process implemented on St Helena is based on the UK Government’s six-phase WSAO guide [16], of which St Helena is currently at phase four. The guide provides a structured approach on how to put a WSAO into practice, including understanding the causes of obesity in local populations and co-producing actions and interventions to reduce population levels of obesity (see Fig. 1). Phase one aims to secure senior-level support and establishes the necessary governance and resource structure to implement the approach. During phase two a compelling narrative is built describing why obesity matters locally and creates a shared understanding of how obesity is currently addressed. Phase three brings stakeholders together to create a comprehensive map of the local system that is understood to cause obesity in what is known as “workshop one”. During phase four, stakeholders come together to prioritise action areas in the local system and propose collaborative and aligned interventions in what is known as “workshop two”. Phase five focuses on maintaining momentum by developing the stakeholder network and an agreed action plan and phase six aims to get stakeholders to critically reflect on the process of undertaking a WSAO and consider opportunities for strengthening the process. St Helena has not yet begun these last two phases.

At the time of this report, the St Helena WSAO was part-way through phase four of the UK Government process as they had delivered workshop two, a key milestone for creating a local system map. The process continues and St Helena are currently drafting the action plan. Therefore, this process evaluation focuses on the first four stages of implementation of the WSAO to learn lessons for implementation and disseminate key learning outcomes promptly.

### Aims

The aim of this process evaluation was to explore the challenges and enablers during the early stages of the WSAO implementation within a small island context and implications for the transferability of the approach to other small island developing states and UKOTs. In theory, this approach might be transferrable to UKOTs because it is designed for local context (small populations), the UK guidance is often used in UKOTs, there is emerging evidence of it working well in local UK contexts supports its use in piloting in UKOTs and there are some similarities in health systems.

Process evaluations seek to understand how complex interventions work. They also seek to understand how well implementation maintained fidelity to the planned approach, how feasible and acceptable the intervention is in the context, and reasons why it may not have worked as intended.

### Technical support

The implementation team in St Helena had limited, if any, experience of whole systems approaches to obesity and limited knowledge about systems thinking and obesity management and prevention strategies. The team in St Helena is also very small with competing priorities. The technical assistance provided by Leeds Beckett University (LBU) aimed to address some of these shortfalls to: build local capacity in systems methods, tools, and approaches; advise and support St Helena with the implementation of the first four-phases set out in the WSA to obesity guide and adapting content to reflect the local context; better understand the local drivers of obesity from a multi-disciplinary approach.

The technical support provided by LBU, included a bespoke training and support package for the implementation team in St Helena consisting of three training sessions which lasted two to three hours each. The training, which took place prior to the main programme workshops, included: (1) an introduction to systems and systems thinking; an overview of WSA and the stages of the UK Government guidance; (2) a practical example of an English WSA to obesity from Oxford City Council; (3) workshop preparation and facilitation; an introduction to systems change; and (4) training on methods and tools to support the process, including qualitative systems mapping, stakeholder mapping, action mapping, and action register completion. Fourteen people attended the facilitator training ahead of workshop one and six attended the facilitator training ahead of workshop two. Attendees included people working in the health promotion team, core working group members, health care and allied health professionals, Ministry of Education staff and retail business owners.

LBU’s support also included facilitation of support from the Oxford City Council’s Public Health team which took the form of two virtual meetings to share learning about their experience of setting up a WSAO and provide peer support when required to the Saint Helena team. During phase three, LBU provided support in the form of reviewing presentation slides used for workshops, virtual presentation of material during the workshops and preparation of systems maps. Throughout the first four-phases of implementation, LBU provided support when needed and virtual attendance at core working team meetings. During the project, St Helena had several COVID-19 related restrictions in place, so activities were undertaken virtually.

### Implementation

St Helena began implementation of phase one of WSAO in 2021 by setting up a core working team (CWT) to undertake the day-to-day operations and coordinate the approach. The CWT was made up of health promotion team members, partners from other sectors (e.g., representative from the equality and human rights commission and a conservation officer), and senior leadership including the Chief Secretary and Health Minister. In early 2022, St Helena worked with the academic team at LBU to collect data to build the local picture of obesity (phase two). St Helena at the time had limited health data available so this stage involved requesting information from partners and making use of what scarce data there was, which often included qualitative data from interviews. Mapping the local system (phase three), brought together stakeholders in a half day workshop to achieve two objectives (1) map out the local system to show how the different factors and interrelated, and (2) begin to develop an overall shared vision for the programme of work. A shared vision is a clear and aspirational statement of what the whole systems approach is trying to achieve. Results of the systems mapping exercise can be found in Appendix 4. Workshop one took place in February 2022 for half a day. Invites were sent to 53 people and 23 attended. As part of phase four, stakeholders were brought together to prioritise areas for action in the local system and propose collaborative and aligned actions. Workshop two took place in May 2022 for half a day. Invites to workshop two were sent out to 46 people and 17 attended. Among the 17 attendees were representatives from the health promotion team, environment and agriculture sector, non-governmental organisations, health care professionals, senior government leaders, social care professionals, Ministry of Education staff and business sector.

### Data collection

A combination of semi-structured interviews, online surveys, and reflections and feedback forms were used to collect data. Semi-structured interviews were chosen because they allowed for detailed information on the views and experiences of people who were involved in implementation of the first four phases of the WSAO. Purposive sampling [19] was used to recruit interviewees to ensure a range of different stakeholders were represented. Purposive sampling has been adopted in interview and survey research previously [20] and thus, this sampling strategy allowed for the recruitment those that had significant involvement in implementation and, therefore, may allow us access to a diverse participant pool and rich data from the quality of data collected. Suitable interviewees were identified by the local health promotion lead in partnership with LBU and OHID (Office for Health Improvement and Disparities). Nine stakeholders were invited to be interviewed and eight interviews were conducted from the following sectors/groups: CWT members; senior partners (e.g., senior stakeholders supporting WSAO); community groups and Non-Governmental Organisations (NGOs); high school students; and local private businesses. Data collection took place between February and November 2022. An experienced qualitative researcher undertook interviews in person which lasted between eight and 29 min *(M*_*duration*_*=21 min*). The semi-structured interview discussion guide (Appendix 1) included 11 open-ended questions, based on the research objectives, structured to prompt discussion with probes and follow-up questions adopted as needed. Questions addressed participants’ expectations and perceived challenges and successes of the process of implementing WSAO so far. Questions were pilot tested with a member of the team in St Helena prior to any interviews, with the content and order subsequently agreed upon by the research team. All data collection locations were free from background noise, where interviewees could not be overheard, in isolated rooms. Interview data were digitally recorded and transcribed verbatim. All data were anonymized to ensure confidentiality.

A range of other sources were used to collect qualitative data to support and validate data collected from the interviews and add further detail including:

- Workshop participant feedback forms (Appendix 2) (paper-based forms distributed at the end of both workshop one and two, designed with the implementation team in St Helena to capture the extent to which expectations were met, what went well and what needed improvement).
- Online stakeholder survey (Appendix 3) on WSAO implementation progress sent out to 47 people including CWT members, people who attended the facilitator training and workshop attendees. The survey was co-designed with the implementation team in St Helena and circulated via email two months after workshop two. The purpose of the survey was to gather a wider set of participants’ feedback (in addition to interviewees) and experiences about the implementation of a whole systems approach so far to better understand the positive and negative aspects.
- Other documentation (minutes from CWT meetings, key informant reflective notes and the LBU end of project report).

Table 1 gives detail to the participants who contributed to this evaluation. In respect of anonymity, especially on a small island, demographics are not provided, and random participants numbers assigned in quotes.

**Table 1:** Participants across data sources

Data source	Participant details
Interviews	Health promotion team members, senior leaders, workshop attendees, core working team members
Workshop feedback form	Workshop attendees (senior leaders, wider partners such as business owners, students, health professionals)
Online Stakeholder survey	Workshop attendees, core working team members, wider partners such as business owners, students, health professionals
Core working team minutes	Core working team members
Reflective notes	Health promotion team lead
End of project report	Leeds Beckett University

### Data analysis

Thematic analysis was used to provide rich, comprehensive, and complex account of data [21] and to allow identification, analysis, and to report on patterns and themes related to the research objectives within the data [22]. Specifically, the six phases to thematic analysis were followed: familiarisation with the data, generating initial codes, theming codes, reviewing themes, defining themes and writing up [22]. First, verbatim transcripts of interviews, survey responses and documents were read and re-read to allow familiarization which, assisted in coding relevant segments of data that addressed our research questions i.e. a theoretical thematic analysis approach rather than an inductive one. Open coding was used to be able to develop and modify the codes during the analysis, rather than pre-set codes. A charting and mapping exercise was then carried out to draw out the main themes and sub-themes (by researcher BM). These themes were developed both deductively, based on the research objectives, and inductively which allowed data to be classified in such a way that was relevant to the research objective, but while maintaining the openness for unforeseen themes to emerge. A second senior researcher (IFW), experienced in qualitative research, oversaw the data analysis process by reviewing the themes, prompting discussing between the two researchers to define the final themes.

### Ethics

The study was approved by St Helena Research Council Ethics committee and included both the implementation work and the research evaluation aspects of this project (institutional ethics reference: 101,068). Informed consent was obtained from all the participants and/or their legal guardians. No vulnerable individuals or those under the age of 16 were recruited.

## Results

The main barriers and facilitators to implementation are summarised in Table 2. and discussed in further details below.

**Table 2:** Landscape summary:

WSAO Phase	Main barriers	Main facilitators
Phase 1	Lack of diversity in CWT. Lack of capacity from CWT members. Limited baseline knowledge of obesity and systems thinking. Internet restrictions meant pre-reading was a challenge. Transient nature of the CWT membership meant new people joined later and needed to be caught up.	Senior level support. Academic support. Peer learning from English local authorities.
Phase 2	Lack of local data on obesity and NCD burden. Stigmatising language.	
Phase 3	More time needed during workshops for contextual/background information. Workforce constraints. Participation fatigue.	
Phase 4	Limited time in workshops to complete activities.	

### Evaluation by phase of process

#### Phase one: set up

Several key factors were identified by participants from all data sources regarding the successful implementation of the initial set up phase of the project, such as the importance of senior level support such as senior civil servants to help progress the early stages of the approach. CWT participants frequently mentioned in interviews the lack of capacity to become a member of the CWT (phase one barrier):

*“We’ve had several different partners who have wanted to be a part of the CWT but have been unable to commit due to other priorities and commitments”* (participant three).

Participants also noted the need to broaden CWT membership to include a more diverse range of people (phase one barrier). One participant explained how a more representative CWT would make it feel more like a community project rather than a government project. One participant suggested having less frequent CWT meetings may have attracted more members.

Although one participant attributed their prior knowledge and experience of using the UK Government’s guidance and implementing a WSA in the UK to helping with the set up and early stages of implementation, this was not the case for all participants. Overall CWT members had limited baseline knowledge and understanding of obesity, public health, and WSAs. Related challenges included internet restrictions meaning pre-reading was a challenge; the transient nature of the CWT membership which meant new people joined later and needed to be caught up; and the public health team having low knowledge and experience levels of systems thinking (phase one barriers):

*“Before initiating a WSA on a small island developing state (SIDS) [UKOT] it would be helpful to assess the knowledge, experience, and capacity of the public health team leading the WSA to identify if preliminary work to support the team’s knowledge and experience in healthy weight and obesity should be completed first”.* (participant nine).

*“Partners, including CWT partners have limited access to the internet and printers. Therefore, sometimes information needs to be printed out for partners and time allocated in meetings for people to read information, that would usually be sent and read ahead of attending a meeting.”* (participant nine).

*“Current and former members [of the CWT] have joined/left at different stages which has perhaps contributed to differing baseline knowledge and understanding of the WSA.”* (participant nine).

Participants found the academic support useful (phase one facilitator):

*“Without the collaboration I think it would have been difficult for St Helena to have established the approach as far as we have, particularly in regard to the practical expertise and experience from LBU and OHID (Office of Health Improvement and Disparities) in having implemented this approach in different areas…. Having the collaboration and expertise has helped to gain interest and engagement with partners across the island. It is seen as an opportunity by partners for St Helena, especially in being part of an academic partnership with LBU and the UK government, but in also being a pilot for UKOTs”* (participant three).

The academic teams’ whole systems and facilitator training was outlined as being helpful in the online stakeholder survey, as it improved confidence and competence in the local workforce. The use of well-chosen locally relevant examples and the theory helped understanding for the whole process and was highlighted as essential in future projects.

LBU’s presentations in workshop one were well received as participants felt their expertise added value to the workshop. However, meeting minutes showed that the CWT felt LBU’s workshop facilitator training was rushed and the academic team felt the technological issues of delivering virtual training meant two-way interaction was extremely difficult, which is an important part of participants understanding concepts that are new to them. One participant from the CWT explained that a wider range of people needed to attend the training.

The opportunity to learn from a local authority in England and how they adapted UK resources for their context was reported as useful, particularly developing the system maps (phase one facilitator). Some participants felt that the timeframes, rather than the amount or type of support, for the academic support contract were too short as its mis-aligned with the normal ways of working on the island which meant that it had a detrimental impact on other health promotion work.

#### Phase two: building the local picture

Fifteen responses were received from the online stakeholder survey (32% response rate). A total of 86% of online survey respondents said that a clear local picture about obesity in St Helena was “fairly well” developed suggesting that the lack of local data that was available for collection limited the ability to accurately and fully describe the burden of obesity in St Helena.

A member of the health promotion team noted in reflective notes that there were challenges in obtaining statistics for obesity and noncommunicable diseases (NCDs) for St Helena and other SIDS/UKOTs (phase two barrier):

*“UKOTs will have differing levels of local health data available to be collated and analysed. This will need to be considered during the early stages of the WSA when collating information to share with stakeholders to help set the local scene in relation to obesity, and when trying to monitor changes*” (participant nine).

Also stigmatising terminology and language was perceived to be a key issue, as noted in reflective notes (phase two facilitator):

*“Consider language used (healthy weight, living with obesity, etc.) as the use of ‘obesity’ was highlighted quite early on by partners, especially from a stigma point of view and partners highlighting that this could disengage people. At several different points people asked what obesity is, so this should be considered when thinking about when engaging partners and when facilitating workshops (UK starting point vs UKOT starting point). Consider cultural norms around body weight/shape and the language used around this. This was highlighted by partners in workshop two and is an important point to consider when empowering, educating, and engaging partners”.* (participant nine).

An addition was made to workshop one activities outlined in the UK guidance [16]. Stations around the room were created, with questions posted on flipchart paper on the walls such as “What Should we Call our Approach?” and “Language we Want to use”. These stations served as an interactive tool which encouraged participants to discuss ideas and were then used as a springboard for discussions in workshop two.

Half of the survey respondents were satisfied with the information and support that was given to prepare and participate in the workshops. Suggestions for further information and support included: system mapping exercises, online talks by health experts on healthy living and systems mapping, information about the academic team’s input into the project, case studies on systems approaches implemented around the world, strengths, weaknesses opportunities and threats analysis of the existing system in St Helena and more information on obesity with specific relevance to St Helena.

#### Phase three: mapping the local system

Responses were received from 18 participants responded to the feedback forms (78% response rate) and 10 participants (59% response rate) for workshop one and two, respectively.

After workshop one, most (90%) workshop feedback survey respondents felt they had a better understanding of how obesity connects with their work. All respondents felt they had a better understanding of systems thinking and how it applies to their work (100%). Most (90%) said that the workshop increased their awareness of the complexity of obesity and the types of actions required to address obesity. However, it was noted by workshop attendees that there was a need for more introductory material to be provided, such as attendee introductions and contextual information (phase three barrier).

Following workshop one, most survey respondents (94%) felt the WSAO process would deliver change. One participant explained that funding from other sources was needed and there was limited budget for stakeholder engagement activities:

*“… we want to promote stuff and we got no funding so that will be a challenge”* (participant six).

Conversely, another participant thought it was encouraging that there was a St Helena Government (SHG) budget for this work and indicated that SHG wants to see change:

*“I think the fact that the government has continued to fund the Health Promotion workers post submission indicating that they want to see change*” (participant seven).

Workforce constraints including sustainability issues of a transient workforce and competing priorities with other health promotion work was a frequently mentioned concern regarding successful delivery of the WSAO (phase three barrier). Concerns around the lack of momentum of the work were also made, partly due to the challenge of competing public health priorities.

*“Three participants out of the eight who were interviewed, alluded to the need to measure progress against more regular, incremental milestones to improve stakeholder and senior level engagement with this work as ultimately it is a long-term approach where impact will not be seen for a while: “… if you’ve been able to see results a little bit quicker, then people might have been able to stay on board with something actually happening rather than just talking about what we’re going to do”* (participant five).

After workshop one, all feedback survey respondents (100%) felt that the time, resource, and work capacity commitments required from them were feasible. Most feedback survey respondents (94%) felt that the WSAO process will help them to engage and collaborate with other stakeholders on the issue of obesity. Participants noted that the opportunities and activities for group thinking were useful in workshop one. Survey feedback indicated that the workshop provided a good opportunity to hear views from a range of different stakeholders from different sectors:

*“…great opportunity to hear from other people from different areas of work and ages”* (participant 10).

All participants explained that a broader range of stakeholders was needed at the workshop (phase three barrier). For instance, the private sector were not represented well as the workshop clashed with a key cargo ship arrival for merchants. This may have resulted in underrepresentation and diversity of workshop attendees. One participant suggested a way to address this issue in the future:

*“I think we went about the approach backwards. We chose people and now we want to promote it, but we should have promoted it and seen who wanted to come with us and then invite people”* (participant six).

92% of respondents agreed that a shared vision had started to develop during workshop one through stakeholders coming together in workshops and agreeing priorities and actions that everyone could take to address the common challenge of obesity. The systems mapping and action plan activities had helped to create this shared vision. All respondents agreed that during workshop one, stakeholders started to effectively map the local system to see where and how they can help to prevent and manage obesity and what they are collectively trying to achieve (i.e., *“a healthier population”*). The final versions of these maps can be found in Appendix 4. The creation and development of these maps were inspired by the Foresight Obesity System Map [23]. During the workshop, as per the UK Government guidance, attendees mapped out local causes and contributors to obesity to see where, and how, they can help to prevent and manage obesity in their own personal and professional fields. Mapping the local system also helped to identify where actions may have the greatest potential leverage. Some respondents mentioned the value of exchanging ideas and views amongst a broad range of stakeholders when developing and agreeing the map of the local system.

Respondents mentioned time constraints of workshop one as something that did not work well during the development and agreement of the map of the local system although they did not specify how much more time was needed (phase three barrier). Respondents felt that four hours was not enough time to work through the map, to add detail and allow all participants to contribute. Furthermore, participants said that the mapping exercise was challenging but useful. Some participants felt more time was needed for attendees to ask questions and perhaps another break. These challenges were illustrated in reflective notes:

*“The system mapping was quite a difficult exercise for many as people were unsure as to which direction the arrows so, I think having trained facilitators at each table really helped. I’d recommend training/run through of the activities for any future islands in this approach, especially as systems thinking is a new way of working for the area, or of it is a small team delivering the workshops. When walking around there were lots of queries, so one lead person walking around the room could not answer all of the questions coming from the tables.”* (participant nine).

Participation fatigue was discussed in slightly different contexts. One participant explained that it is often the same people on island that get asked to participate in projects such as this which can result in participation fatigue but also means that some people may be being consistently missed out in these types of projects (phase three barrier):

*“With a smaller population and workforce on a SIDS/UKOT, there are less people available to be surveyed and request information from. Teams leading the WSA should be mindful of survey fatigue and novel approaches to data collection may be required, especially in communities with limited access to the internet and e-mail”* (academic partner).

There were practical issues in the delivery of the workshop, such as not enough expert leads and trained facilitators to facilitate the discussions. Generally, there was positive feedback on the pace, planning, delivery, and informative content of the workshop. Most survey respondents said the workshop’s activities were above or met their expectations. Furthermore, interviewees said that the information in the workshops was easy to understand, despite the complex nature of the work. A key learning identified was to provide a more in-depth introduction to the topic of obesity and to introduce all attendees to each other at the start of the workshop. Participants said that the main outcome from workshop one was around awareness of the issue of obesity.

One participant explained that the templates in the UK Government implementation guidance were useful, however others described some examples of local context adaptions were needed. In addition to the need to use local examples in training and presentations, the importance of using culturally appropriate language when talking about obesity was stressed, for example, the term “obesity” and “overweight” was considered offensive and stigmatising:

“*the use of “obesity” was highlighted quite early on by partners, especially from a stigma point of view and partners highlighting that this could disengage people”* (participant three).

#### Phase four: action planning

The aim of this phase was for stakeholders to refine the shared vision and to propose actions that may provide the greatest opportunity to change the system. A facilitated workshop (workshop two) helped participants with this process. While an action plan has not yet been agreed at the time of this early phase process evaluation, most survey respondents (80%) felt they had a better understanding of how obesity connects with their work and that the process will help them engage and collaborate with other stakeholders about obesity (90%) after workshop two. Most respondents (90%) also felt they had a better understanding of how the WSAO on St Helena will operate and how it will deliver change (80%).

After workshop two, most respondents (70%) felt that the time, resource, and capacity commitments required from them were feasible. However, many (75%) noted that there was not enough time in workshop two, especially for feedback from the activities, networking, and reviewing the maps (phase 4 barrier). This was echoed in interviews by the team leading the implementation on St Helena. One key enabler of the workshop that was noted was the senior level support shown by the attendance of the Minster and Chief Secretary.

Whilst LBU noted that most of the preparation for the workshop was carried out in-line with the UK guidance, some additions were made to the content. An additional activity was added to review and provide feedback on the system maps:

*“Adding the activity to review and provide feedback to the local system map was a very valuable inclusion to the workshop agenda. It provided an interactive opportunity for the CWT and LBU team to show the development of the local system map and for the attendees to see how their initial input had been developed. The review of the system map enabled further discussions about the local system to happen, that had not been made at workshop one, e.g., using other priorities (food security and climate change) to engage and initiate change”* (participant nine).

All participants said they would recommend implementing a WSAO for other UKOTS:

*“I would say just go for and get stuck into it and take advantage of the opportunity”* (participant four).

#### Phases five and six: next steps in implementation

At the time of this report, St Helena had not yet begun phases five and six, however participants shared plans and proposed suggestions for how the WSAO would progress during these final two phases. For example, participants discussed the need to develop a clear stakeholder engagement strategy to spread awareness and knowledge about obesity causes and consequences amongst the St Helena community and use consistent messaging. Getting the community involved in what type of messaging would resonate locally is a key consideration for the future steps of implementation. One participant explained that it was important to embed this work into other public health initiatives to align efforts:

“*The limited capacity of the CWT and the lack of budget potentially makes the sustainability of the project a risk. It is therefore important when identifying possible actions, strategies, and policies that they tie in with local priorities (e.g., climate change, food security). This will help ensure the work is embedded and sustainable.”* (participant nine).

A number of wider issues were also raised by participants that may be pertinent to the approach going forward and are outlined in Table 3 below.

**Table 3:** Wider issues raised about the future of WSAO in St Helena

Wider issues raised	Quotes
The need for “on the ground” experience to support the CWT	“ [There was a] l*ack of resources in the sense of specialists on the ground that are fully dedicated to that because comparing it to the oxford team and our core working team, I mean they were all experts” (participant eight)”*
Clarity was needed on expected outputs	“*What happens after the [action] plan and how will it continue to be voiced and consistent after the end of the plan?*” (Survey responder)
Some felt it was not yet feeling like a community approach	*“It’s not only St Helena Government’s responsibility, it needs to become a community thing”* (participant eight)
Participants acknowledged that there were unknown consequences of the new internet sea cable coming in the near future	*“Considerations about changing the narrative and awareness about when the cable will land/ arrive. It’s an interesting transition period.”*(participant nine)
The sheer number of big issues to overcome in St Helena such as restricted food supply which could affect the successful implementation of a WSAO	*“and then start to think about the big problems like there’s probably not a lot of gyms or like import and export, and with the food [supply]….So you kind of see the bigger picture”* (participant four)

## Discussion

Overall, the early stages of the WSAO were successfully adapted and implemented on this small island. All participants were supportive of the approach and recommended it to other small islands to adopt. Awareness and understanding of obesity and whole system approaches grew and there was reasonably good engagement across most sectors. Several concerns were expressed about the continuation of the approach to lead to multi-sectoral action.

### Key findings in the context of existing evidence

1. Local context and adaptations to the implementation guide

The suggestions made for further information and support that would have been helpful in advance of the workshops, mirror the insights from Halton Borough Council’s pilot which showed ideas to help familiarise new attendees with the process up until that point by providing a written overview of workshop one [16].

As part of the stigmatising language issues that arose, the name of the WSAO was also highlighted as something that would need to be changed for St Helena as workshop attendees felt it was important to contextualise the work and not include “obesity” in the title. A similar issue was mentioned by Gloucestershire County Council, a pilot site for the WSAO. Community insight from Gloucestershire County Council showed that families would not engage with the planned “Food and Health” project but changing the name to “Food and Families” made the project more relevant to the community, improving engagement [16].

These are encouraging findings as they suggest that there are not significant barriers to implementation of the UK Government’s guidance to a WSAO in small islands and with existing guidance and expert support, early stages of successful implementation are possible.

Whilst this process evaluation on WSAO is valuable for small island contexts in particular, more should be done on other WSAO projects around the world to gain practical evidence of implementation.

2. Senior level support and stakeholder engagement

Strong leadership for the approach from the health promotion team and from senior civil servants and politicians was recognised by interviewees and appeared to be the main aspect that has been working well so far.

The importance of strong and trusting relationships between multi-sector stakeholders within whole systems approaches are stressed in the UK implementation guidance especially for creating a sustainable foundation to encourage community ownership of the approach [16]. Work that is co-produced and an approach that works closely with local people will help to successfully deliver change [24]. This process evaluation found evidence that key stakeholders and community members have been identified and participated in the early phases of the approach. Insights from the London Borough of Lewisham and Oldham Council’s pilot showed having a senior representative present made stakeholders feel listened to and stressed the importance of the issues being discussed. Efforts to build strong relationships with the private sector and wider community are needed as this is considered a key aspect to influencing effectiveness [25]. Participants from the health sector talked about the need to engage people who don’t normally get involved with projects such as this. Engaging these non-traditional partners such as community champions can help to disseminate messages and actions [26].

It was recommended by a member of the health promotion team that other UKOTs should conduct early community engagement before the workshops. Doing so promotes the project to the community first, indicating who is interested in being involved and this may lead to increased workshop attendance and future community engagement. This aligns recommendations made by partners in a similar process evaluation in Australia to “engage community members first through assets they provide for community action, not agencies or organisations they represent” [27].

Common features of successful multi-level, community wide interventions reported in process evaluations, and deemed key to building a successful WSA, included engagement of partners and community; time to build relationships, trust, and capacity [13, 17, 27]. Engaging communities in a WSA is a key implementation element. Participants from the CWT talked about how stakeholder engagement momentum has been lost over time and there is a need to maintain this community interest as well as keeping senior leadership team engaged. Participants from the CWT explained the challenge of partner commitment issues and whilst they may have interest in the work their capacity to be involved is limited. These are a similar finding to Scotland’s process evaluation of early adopters, where stakeholder engagement has been difficult because of limited capacity to be involved and difficulties encouraging certain sectors to recognise their role and influence [18]. Maintaining stakeholder engagement will be challenging as WSAO is a long-term initiative and significant impact on population weight status is unlikely to be seen for several years. The importance of recognising that the WSAO is an iterative approach and no immediate results seen was highlighted by participants. Instead, long-term change will be the impact and success of the approach.

Stakeholder fatigue has also been found to be an issue during the implementation and was experienced due to the demands the stakeholders experienced, particularly due to the frequency of the meetings stakeholders were required to attend. This was cited as a reason for limited stakeholder engagement in Scotland [18] and, therefore, is something that future WSAs, especially in small islands, need to be mindful of when working with stakeholders.

3. Capacity issues affecting implementation

Participants from the health promotion team discussed the starting point for the workforce and stakeholder’s baseline understanding of obesity and public health was lower than that typically of a local authority in England. This is an important consideration when implementing such an approach on St Helena and should be addressed through improved communications and information sharing with stakeholders to develop their understanding of the issue and the approach. Some suggested topics to improve on are listed in the local context and adaptations section. In Scotland, communications activities before workshops were used to “warm [stakeholders] up to the WSA process” as well as informal sessions to shape stakeholder’s expectations of the process and provide further information [18].

Limited capacity of a small workforce was also noted as an implementation challenge, especially as unexpected situations such as the COVID-19 pandemic impacted heavily on the team’s small resource, and it also had knock on effects of pausing other health promotion related work as there was a time pressure of LBU’s contracted support. As found in St Helena, other SIDS and UKOTs typically have small populations and workforces can be more transient compared to larger countries. However even in Scotland, communities struggled with staff capacity to deliver the process as the time required for preparing and delivering a WSA was an extensive commitment, especially on local leads and administrative support but also on CWT members and wider partners [18].

The difficulties of establishing the project because of workforce limitations (e.g., small team, limited baseline knowledge, and competing work priorities) were mitigated somewhat by the technical support provided by LBU and OHID such as taking on some tasks from the local team. However, maintaining sustainability with a limited and transient workforce and population was a concern of many of the participants. Adaptations to the structure of the CWT and the networks that surround the CWT are needed to ensure knowledge and labour are spread broadly across a wide network of individuals and organisations. These human resource factors are common to other complex implementation experiences [28].

4. Infrastructure

The nature of St Helena being a small island was described as being an opportunity for success as there is potential for this work to reach across the whole population through existing communication channels like radio which has a very large audience, and word of mouth, through a closely knit community. There is also opportunity for strong measures and levers to be used such as fiscal and legislative measures to the food and built environments.

### Limitations

Limited venue space, unreliable internet, and limited equipment availability meant logistics for CWT meetings took more time to coordinate which had a knock-on effect on speed of implementation and on the delivery team’s workload.

A key limitation of the academic support was that it was all virtual/remote due to the COVID-19 pandemic restricting travel. Two-way interaction was difficult and made it harder for participants’ understanding new and complex concepts and the unreliable internet connection in St Helena interfered with this support offering. Prior research notes the superiority of in-person attendance and interactions in increasing local understanding and building rapport [16]. This is in accordance with participants’ views that physical attendance from the academic team would have been really valuable, particularly during the workshops.

Limitations of this evaluation included that only a small sub-sample of stakeholder groups were recruited. This, along with a lack of representation of some community members, means that the results may be limited in their generalisability. The self-reported nature of the data are also a limitation, as is the presence of self-selection bias which resulted from the sampling methods adopted. There was a relatively large variation in interview length (between 8 and 29 min) which suggests that certain interviewees provided more detail and thus insightful in responses, perhaps because some were more involved in implementation compared to others.

This process evaluation was conducted by staff who had been involved in some of the support provided to St Helena. Therefore, it was not an independent evaluation. However, the research team was not heavily involved in the early implementation processes as most of the technical expertise and support was provided by LBU and other staff with specific WSAO experience. This process evaluation was conducted during the first phases of implementation. A second process evaluation should be conducted during the next phases of implementation to ensure adaptations, challenges and enablers are reflected to contribute to the evidence for and support other SIDS/UKOTS with their implementation of WSAO.

## Conclusions

Early stages of implementation of a WSAO in a UKOT can be successful when using the UK’s resources as a guide and adapting them to the local context, providing academic and expert support to the local workforce and securing senior leader support and participation. This process evaluation has identified risks to the future stages of implementation and sustainability of the approach. Dedicated leadership, securing appropriate budget, and comprehensive stakeholder engagement plans are needed to drive the future stages of implementation. There is an urgency to embarking on the next stages as there is risk of the work losing momentum, stakeholder interest and senior buy-in. It is recommended that another process evaluation takes place once St Helena implements the remaining phases (five and six) included in the UK WSAO guidance to identify lessons learned for this approach in small island settings.

### Electronic supplementary material

Below is the link to the electronic supplementary material.

Supplementary Material 1

## Data Availability

The datasets used and/or analysed during the current study are available from the corresponding author on reasonable request.

## Abbreviations

WSAO: Whole systems approach to obesity
UKOT: United Kingdom Overseas Territory
LBU: Leeds Beckett University
OHID: Office of health improvement and disparities
SHG: St Helena government
SIDS: Small island developing states
CWT: Core working team

## References

[1] World Health Organisation. Obesity and overweight 2021. https://www.who.int/news-room/fact-sheets/detail/obesity-and-overweight.

[2] St Helena Government. Housing & Population Census 2021. 2021.

[3] Centre NI. Health Survey for England 2010: Trend Table 2010.

[4] Fontaine KR, Redden DT, Wang C, Westfall AO, Allison DB (2003). Years of life lost due to obesity. JAMA.

[5] Lauby-Secretan B, Scoccianti C, Loomis D, Grosse Y, Bianchini F, Straif K (2016). Body fatness and Cancer — viewpoint of the IARC Working Group. N Engl J Med.

[6] Guh DP, Zhang W, Bansback N, Amarsi Z, Birmingham CL, Anis AH (2009). The incidence of co-morbidities related to obesity and overweight: a systematic review and meta-analysis. BMC Public Health.

[7] Luppino FS, de Wit LM, Bouvy PF, Stijnen T, Cuijpers P, Penninx BWJH (2010). Overweight, obesity, and Depression: a systematic review and Meta-analysis of Longitudinal studies. Arch Gen Psychiatry.

[8] St Helena Government. Summary of St Helena’s Joint Strategic Needs Assessment 2022.

[9] Government SH. Strategic Framework for Health Promotion on St Helena (2018–2019). 2018.

[10] St Helena School Nurses. and UK National Childhood Measurement Programme.

[11] Haire-Joshu D, Tabak R (2016). Preventing obesity across generations: evidence for early life intervention. Annu Rev Public Health.

[12] Rutter H, Savona N, Glonti K, Bibby J, Cummins S, Finegood DT (2017). The need for a complex systems model of evidence for public health. Lancet.

[13] Bagnall A-M, Radley D, Jones R, Gately P, Nobles J, Van Dijk M (2019). Whole systems approaches to obesity and other complex public health challenges: a systematic review. BMC Public Health.

[14] Local Government. Health in all policies: a manual for local government 2016. https://www.local.gov.uk/publications/health-all-policies-manual-local-government.

[15] World Health Organisation. WHO acceleration plan to stop obesity. 2022.

[16] Public Helath England. Whole systems approach to obesity: a guide to support local approaches to promoting a healthy weight. 2019.

[17] Public Health England. Whole systems approach to obesity programme: learning from co-producing and testing the guide and resources. 2019.

[18] Public Health Scotland. Whole systems approach (WSA) to diet and healthy weight: early adopters programme process evaluation. 2022.

[19] Patton MQ. Qualitative research & evaluation methods: integrating theory and practice. Sage; 2014.

[20] Thorogood N. Qualitative Methods for Health Research. London: SAGE Publications Ltd; 2018. http://digital.casalini.it/9781526448804http://digital.casalini.it/5018381.

[21] Clarke V, Braun V (2013). Teaching thematic analysis: overcoming challenges in developing strategies for effective learning. Psychol.

[22] Braun V, Clarke V (2006). Using thematic analysis in psychology. Qualitative Res Psychol.

[23] Science, GOf. FORESIGHT Tackling Obesities:Future Choices –Obesity System Atlas. 2007. 10.1111/j.1467-789X.2007.00344.x 17316292

[24] Potts A, Shearn K, Frith G, Christy E (2021). Working with local people as part of a whole-systems approach to physical activity: reflections from local delivery pilots. Perspect Public Health.

[25] Guariguata L, Rouwette EA, Murphy MM, Saint Ville A, Dunn LL, Hickey GM et al. Using Group Model Building to describe the system driving unhealthy eating and identify intervention points: a participatory, Stakeholder Engagement Approach in the Caribbean. Nutrients. 2020;12(2). 10.3390/nu12020384 PMC7071222 32024025

[26] Lieberman L, Diffley U, King S, Chanler S, Ferrara M, Alleyne O (2013). Local tobacco control: application of the essential public health services model in a county health department’s efforts to put it out Rockland. Am J Public Health.

[27] Jenkins E, Lowe J, Allender S, Bolton KA (2020). Process evaluation of a whole-of-community systems approach to address childhood obesity in western Victoria, Australia. BMC Public Health.

[28] Lazo-Porras M, Liu H, Ouyang M, Yin X, Malavera A, Bressan T (2022). Process evaluation of complex interventions in non-communicable and neglected tropical diseases in low- and middle-income countries: a scoping review. BMJ Open.

