# Supplementary material for: Process evaluation of implementation of the early stages of a whole systems approach to obesity in a small Island
Source: BMC Public Health. 2024 May 22;24:1376. doi: 10.1186/s12889-024-18876-1 (PMC11110233; doi:10.1186/s12889-024-18876-1)
Supplement: Supplementary file 1 — Supplementary Material 1 [file 12889_2024_18876_MOESM1_ESM.docx]

**Supplementary material**

**Appendix 1: Interview guide**

Process evaluation for whole systems approach to obesity in St Helena Interview Schedule

Interviewer introduces themselves: Explains they are from OHID but have not played an active role in the project implementation.

Introduction: I’m going to ask you some questions about the system approach work supported by Leeds Beckett University. Please be honest and open with all the responses. Just a reminder that they’ll remain anonymous. We’re interested to know from your point of view the elements that have gone well and those that could have gone better.

Expectations

1. The first question I want to ask is about your initial expectations from this work. Thinking back to the start, can you remember what your initial expectations were as to what the systems approach would entail? Have these expectations changed throughout the project?
2. To what extent do you feel you were able to undertake the process as planned?

*prompts: were there specific barriers that affected your involvement?*

1. To what extent do you feel the guidance and support you have been given to implement this work has been relevant for your local area? *prompts: what adaptations have been required of this process? Does the logic model in the guidance document still hold true for a St Helena context? What changes in the approach are needed to enable it to have maximum impact in the UKOTs?*
2. When you think about your mindset, and that of your colleagues’, what is it like now, in contrast to the beginning of the process? Has it changed? If so, how… If not, why do you think that is the case? *Prompts: Is the momentum being sustained?*

Challenges

1. What do you foresee to be the principal barriers to moving the work forward? *Prompts how will the success of the approach be assessed?* *How will learning be captured and shared and with whom?*
2. What personal strengths did you draw on when implementing the process?

*prompt: how did these strengths aid the implementation?*

Core working group.

1. To what extent do you feel that the core working group includes a good mix of representatives across the local system, beyond those working in public health? *Prompts Who declined the invitation to the workshop, who did not end up attending and who wasn’t invited – and why.*

Monitoring

1. What processes do you know about are in place or being developed to monitor and evaluate the WSAO action plan?

Success of the process

1. What have been the main outcomes of this process for you?

*prompt: how did the approach help bring about these changes?*

1. How successfully do you think the project engaged with stakeholders/sectors that were not previously involved?

*prompt: if yes - Has there been a benefit to engaging with these wider stakeholders?*

1. Finally, I’d like you to reflect on where you are at developing a whole systems approach to obesity. A fully operational whole systems approach would involve a shared understanding of the issues by all relevant stakeholders and working together in an integrated way to bring about sustainable change. If you imagine yourself along a continuum where 0 is the beginning of the whole systems work, and 10 is a fully operational whole systems approach, where would you position yourself now?

*prompt: please, expand on why you place yourself there and what you’d need to do to move yourself higher up, say 9 or 10...*

**Appendix 2. Workshop feedback forms**

**Workshop one**

| **Questions** | **Strongly Disagree** | **Disagree** | **Neither Agree nor Disagree** | **Agree** | **Strongly Agree** | **N/A** | **Total No of Areas Replied** |
| --- | --- | --- | --- | --- | --- | --- | --- |
| I have a better understanding of how obesity connects with me |  |  |  |  |  |  |  |
| I have a better understanding of systems thinking and how it applies to my work |  |  |  |  |  |  |  |
| The WSA process will deliver the expected change. |  |  |  |  |  |  |  |
| The time, resource and capacity commitments required from you are feasible and meet my expectations. |  |  |  |  |  |  |  |
| The workshop increased my awareness of the complexity of obesity and the types of actions required to address obesity. |  |  |  |  |  |  |  |
| The WSA process will help me to engage and collaborate with other stakeholders on the issue of obesity. |  |  |  |  |  |  |  |

**1. How could today's workshop be improved for the future?**

**2. Is there anything that you will go away and do as a result of today's workshop?**

**3. Please provide some detail on how today's activities aligned with your expectations?**

**4. Please use the box below to provide any other comments about today's activities.**

**Workshop 2**

| **Questions** | **Strongly Disagree** | **Disagree** | **Neither Agree nor Disagree** | **Agree** | **Strongly Agree** | **N/A** | **Total No of Areas Replied** |
| --- | --- | --- | --- | --- | --- | --- | --- |
| **1. I have a better understanding of how obesity connects with my work.** |  |  |  |  |  |  |  |
| **2. I have a better understanding of how the WSA on St Helena is/will operate.** |  |  |  |  |  |  |  |
| **3. The WSA on St Helena will deliver the expected change.** |  |  |  |  |  |  |  |
| **4. The time, resource and capacity commitments required from me/my organisation, for the WSA on St Helena are feasible and meet my expectations.** |  |  |  |  |  |  |  |
| **5. The WSAO process will help me to engage and collaborate with other stakeholders on the issue of obesity.** |  |  |  |  |  |  |  |
| **6. A shared vision was successfully developed and agreed in this workshop.** |  |  |  |  |  |  |  |
| **7. This workshop was successful in drafting prioritised local actions.** |  |  |  |  |  |  |  |

**8. What worked well when drafting the prioritised local actions during the workshop?**

**9. What worked less well when drafting the prioritised local actions during the workshop?**

**10. How could today's workshop be improved for the future?**

**11. Is there anything that you will go away and do as a result of today's workshop?**

**12. Please provide some detail on how today's workshop aligned with your expectations?**

**13. Please use the box below to provide any other comments about today's workshop.**

**Appendix 3. Online Stakeholder survey**

Stakeholder network survey - St Helena Whole Systems Approach to Obesity (WSAO)

The survey will take approximately 4 minutes to complete.

This survey will be part of an evaluation of the whole system approach to obesity in St Helena.


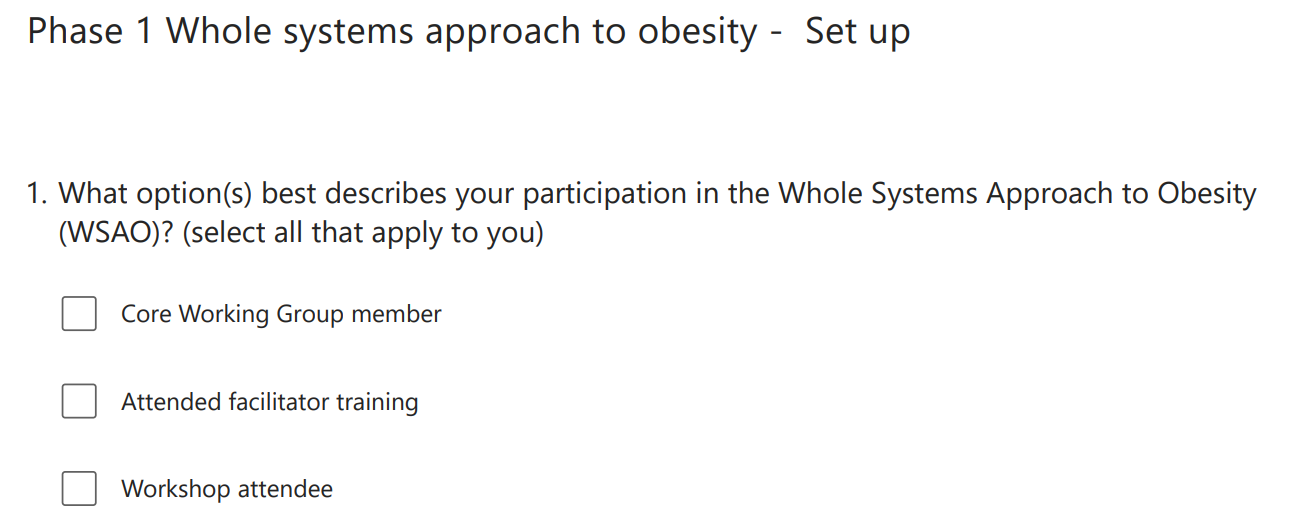

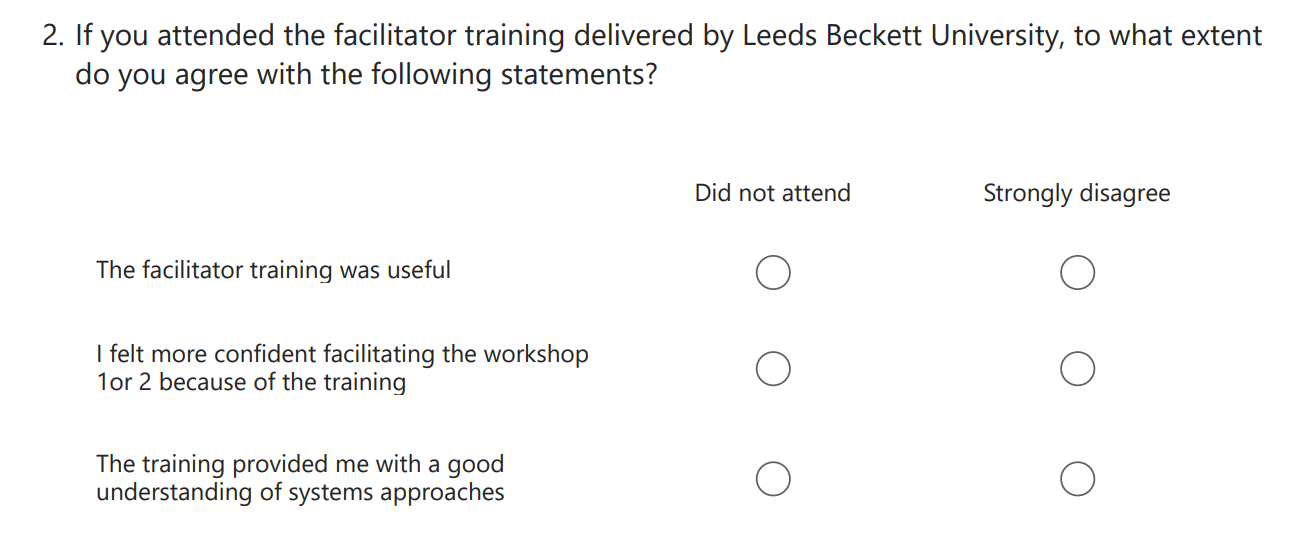


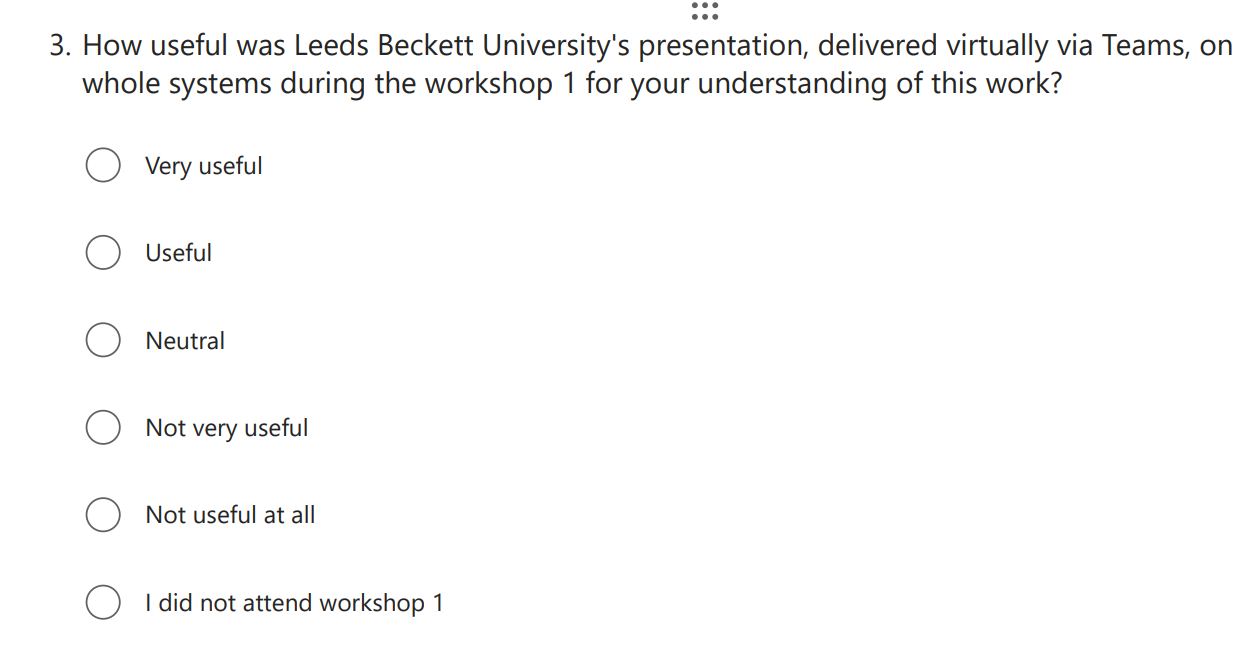

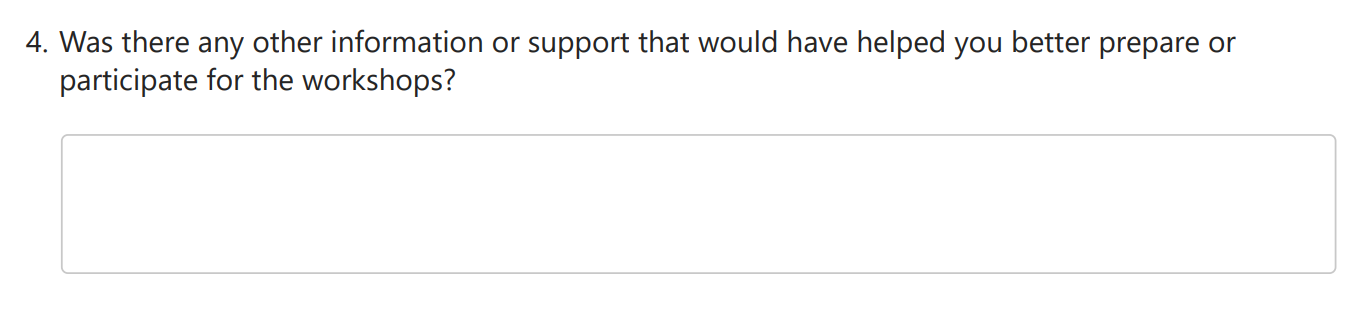

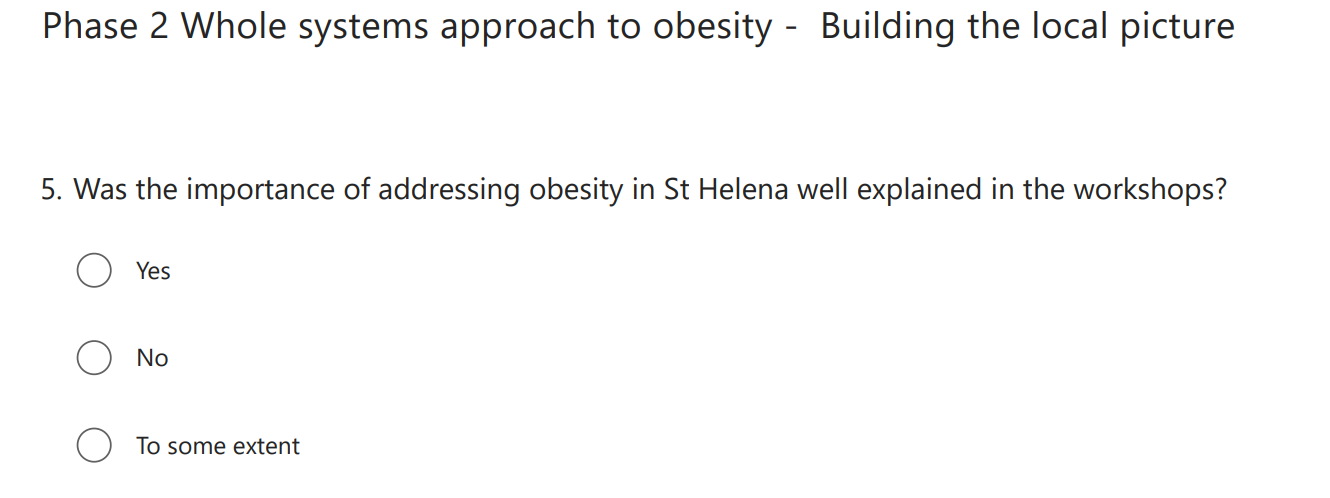


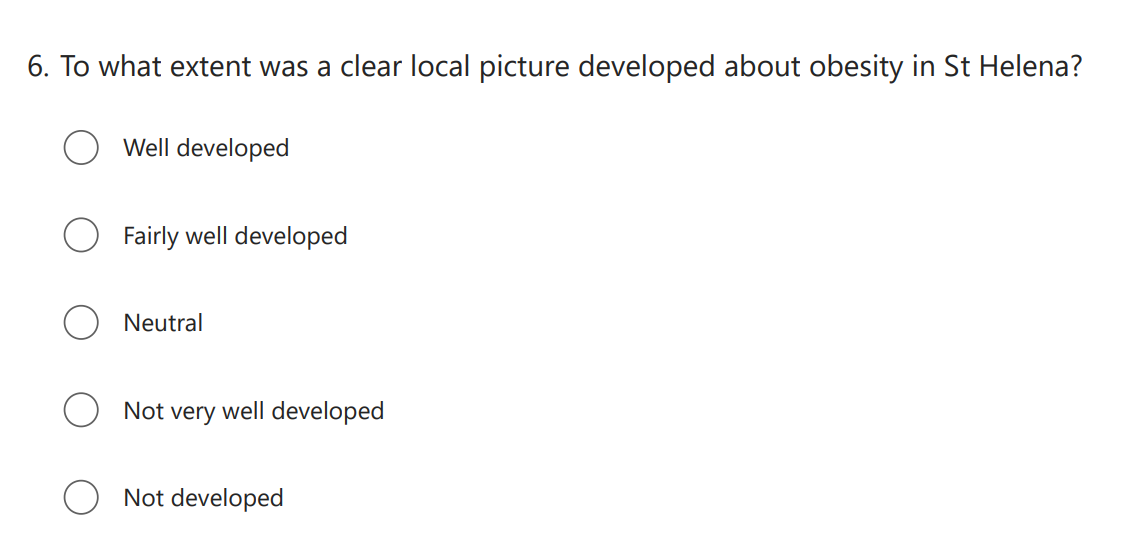

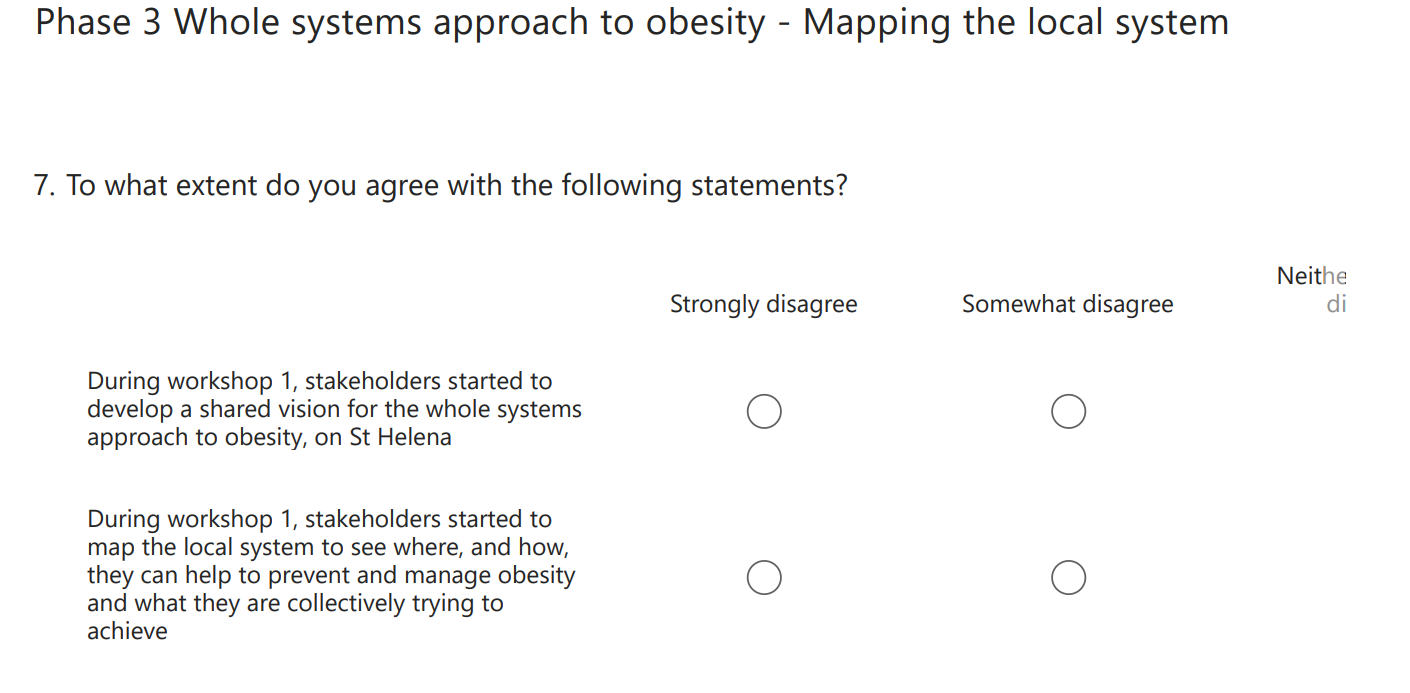

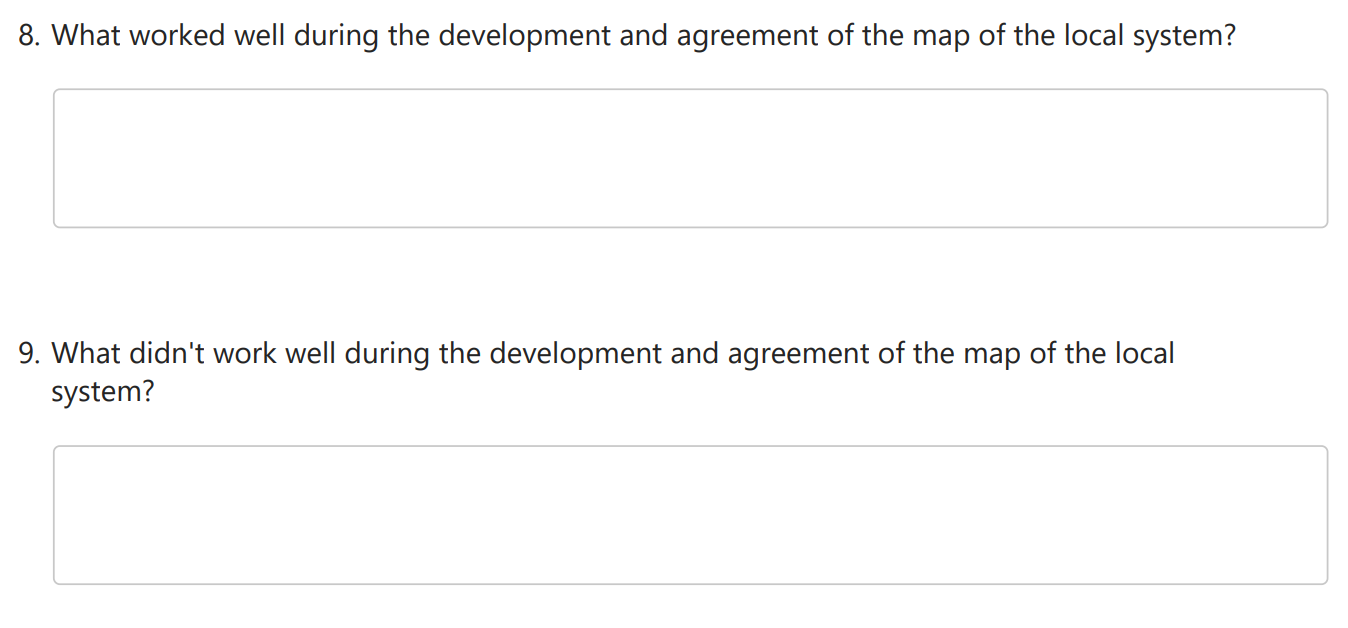


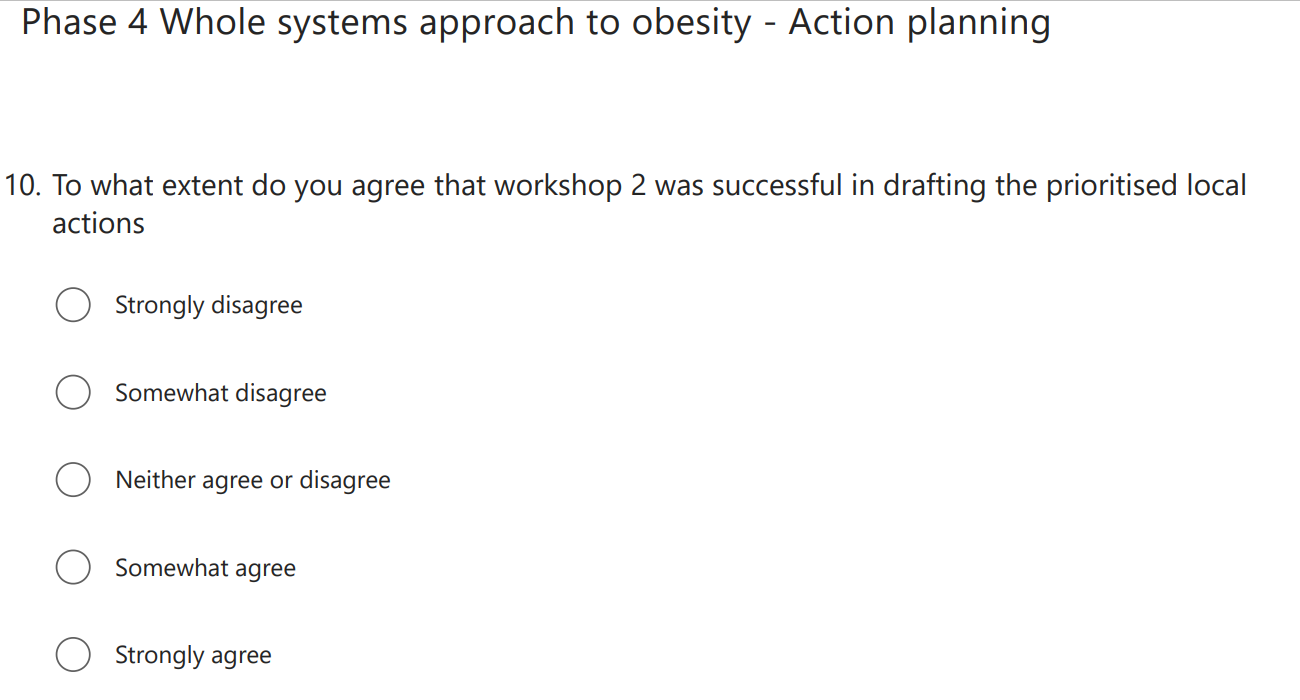

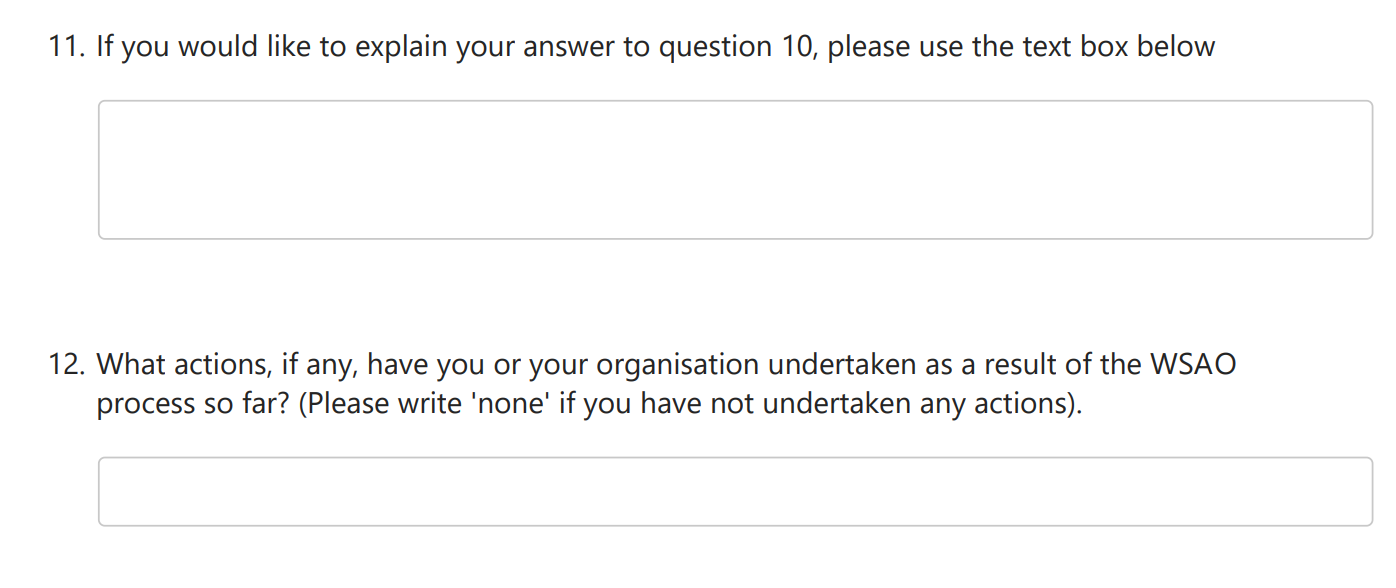

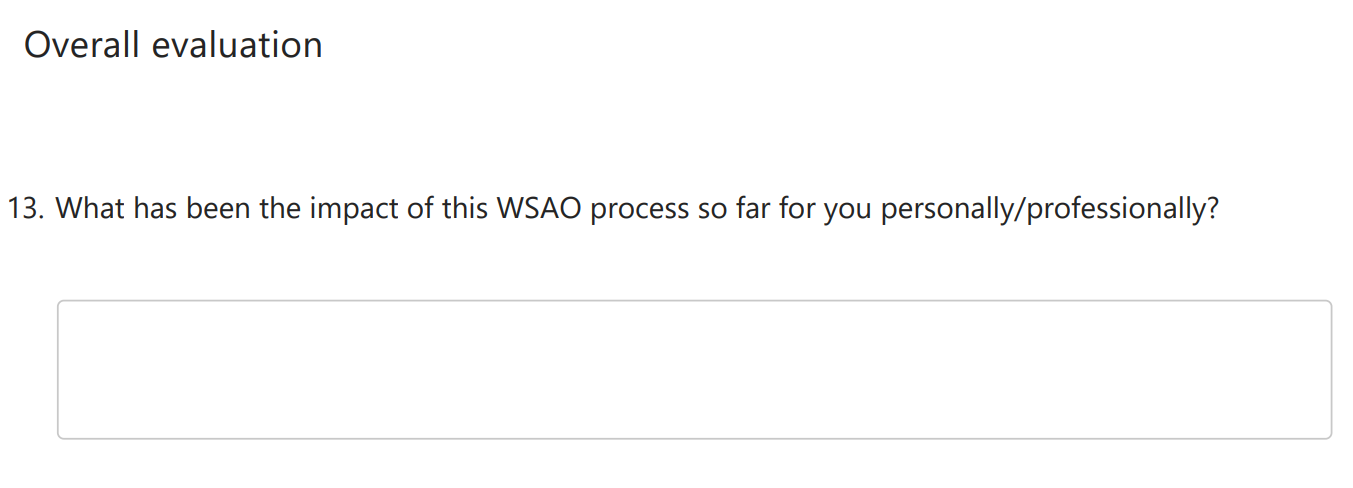


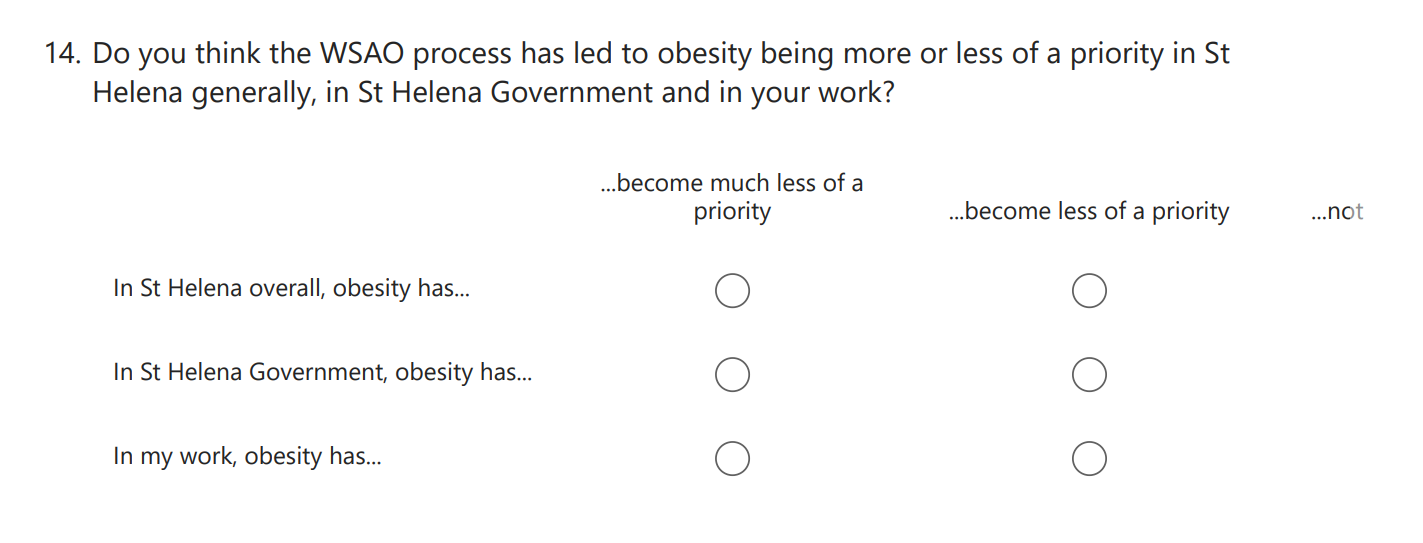

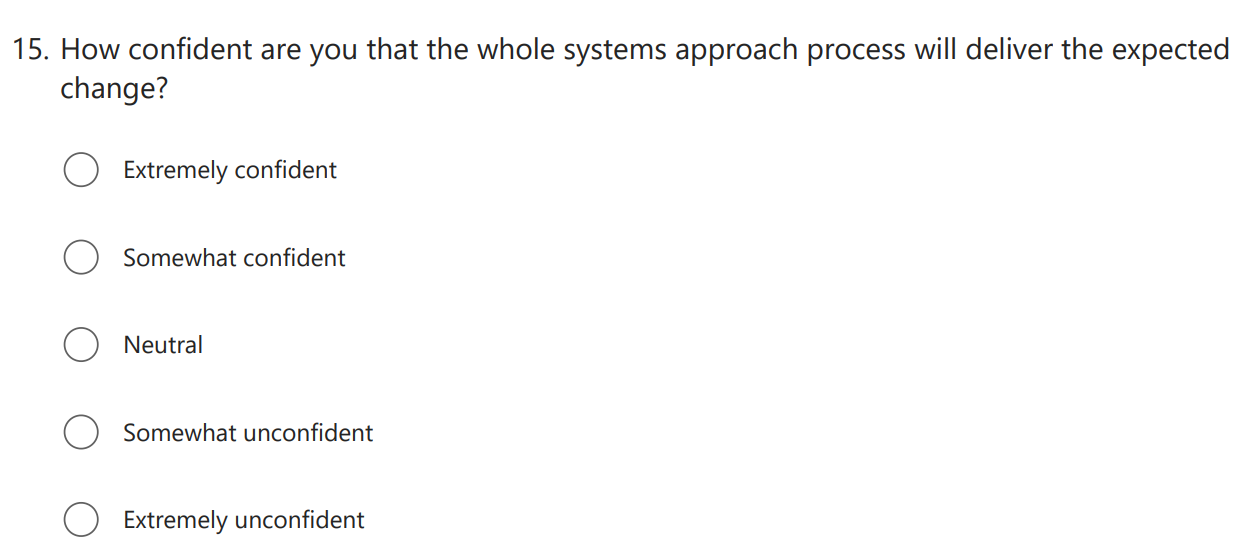

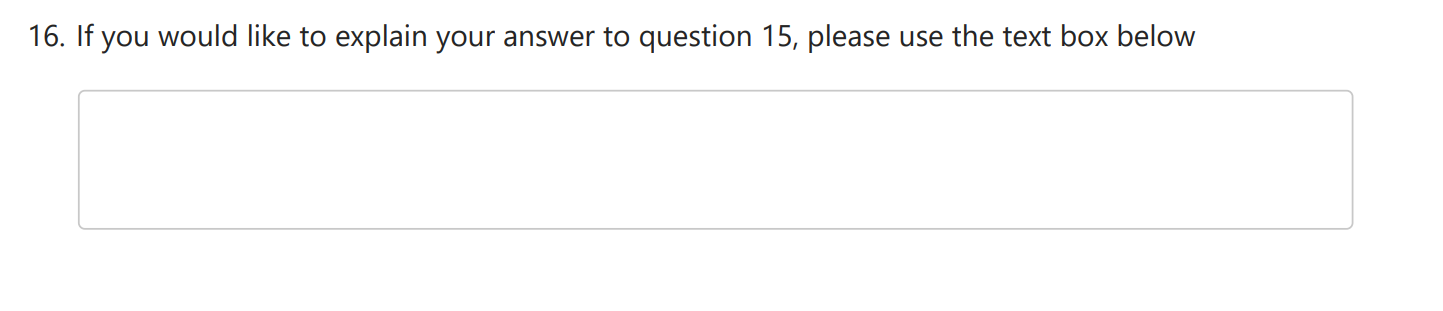


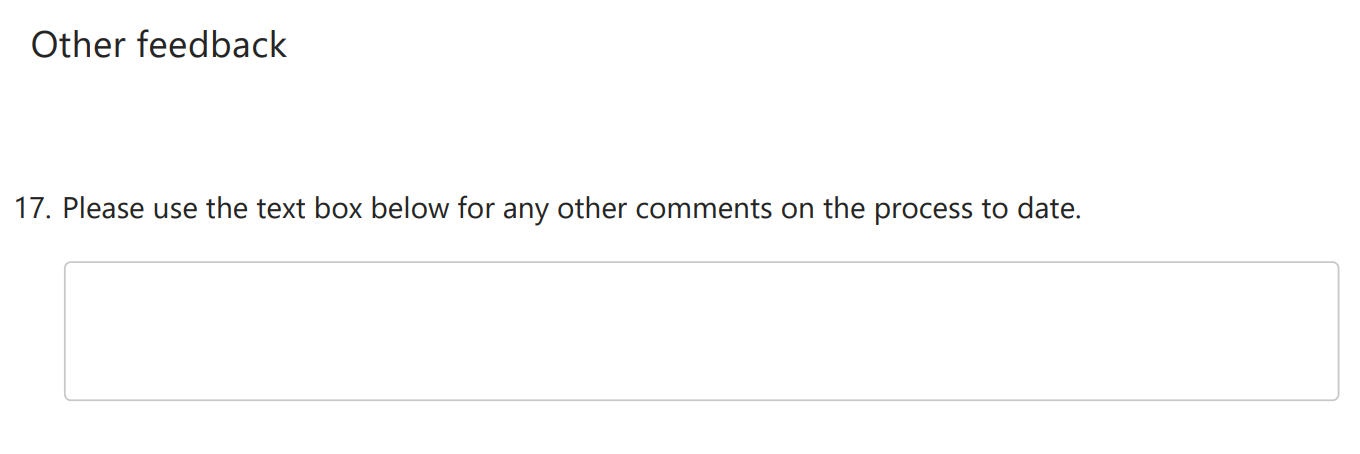


**Appendix 4. Systems maps created from workshops**

**
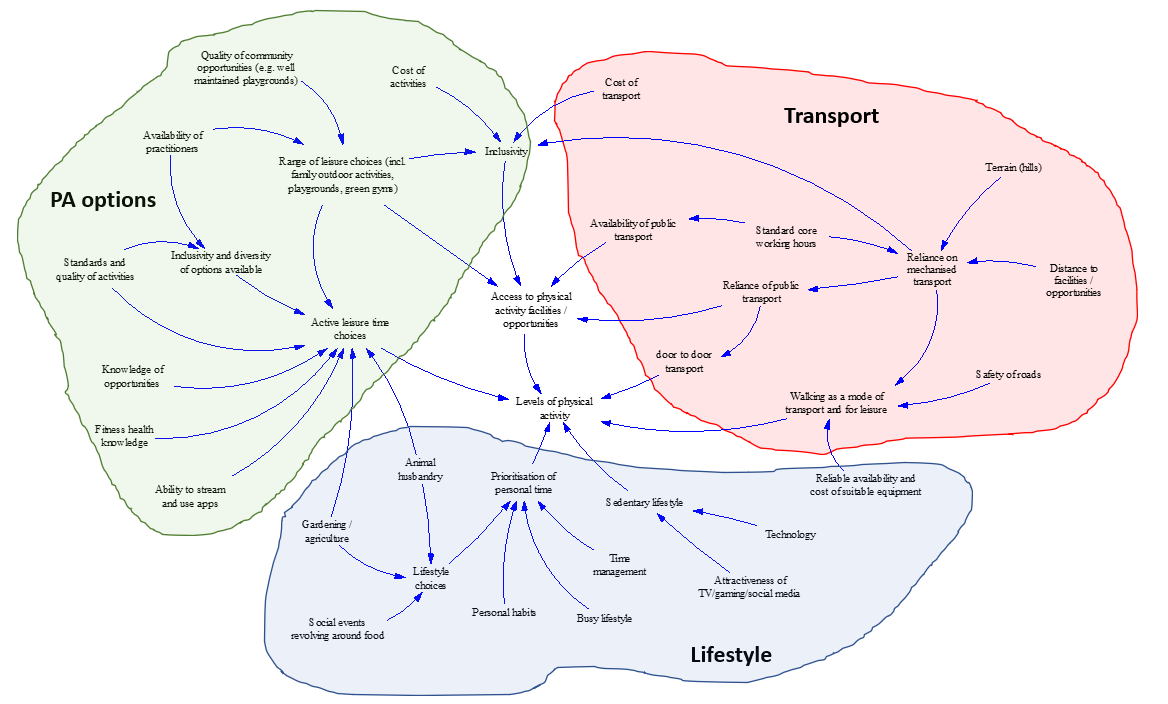
**

**
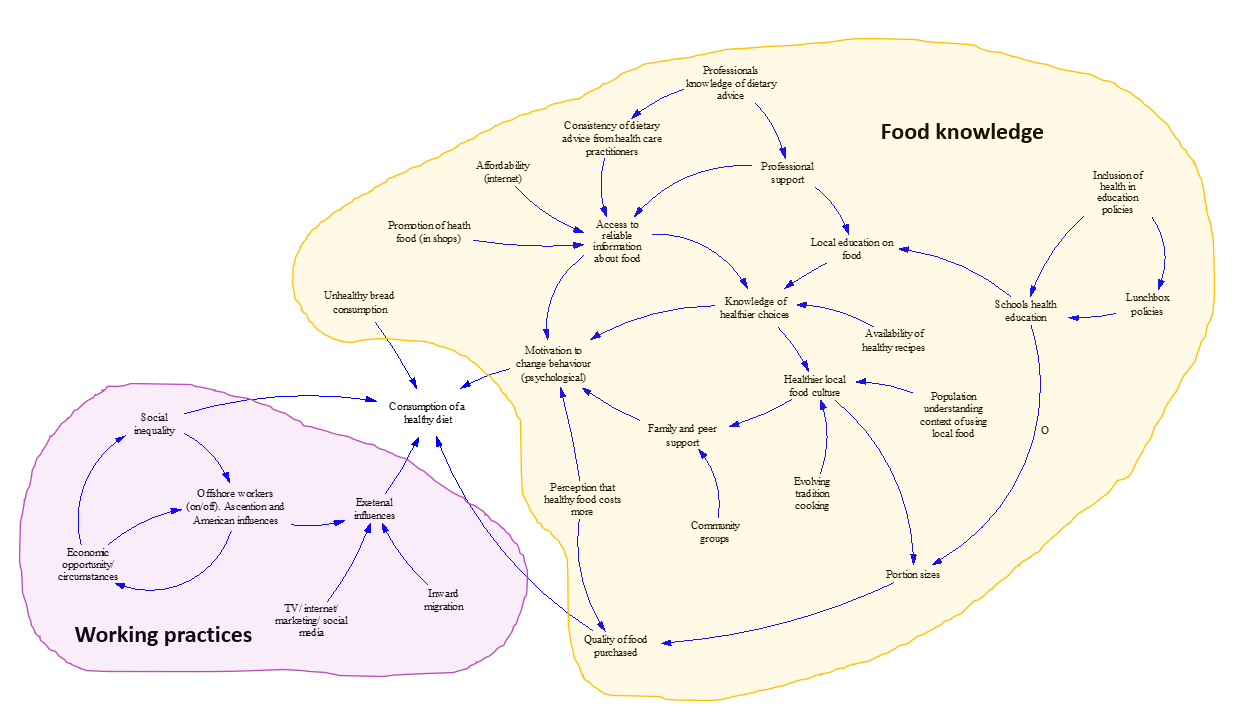
**

**
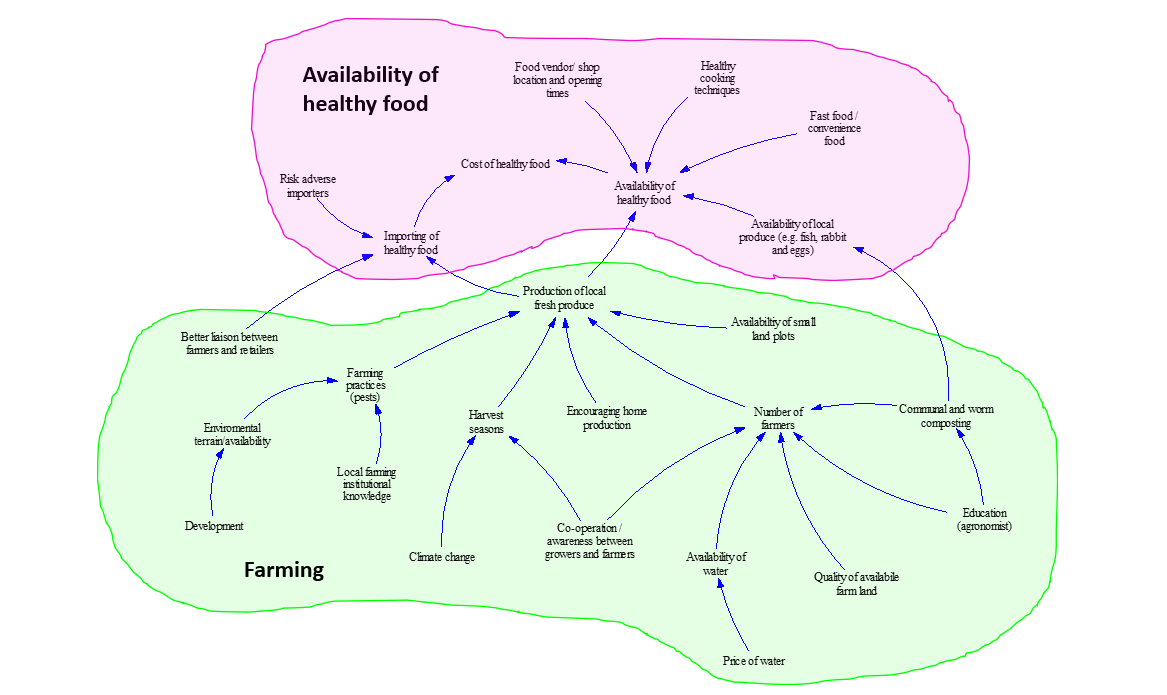
**
